# Insufficient Ratio of Long-Lasting Insecticidal Nets to Household Members Limited Universal Usage in Western Kenya: A 2015 Cross-Sectional Study

**DOI:** 10.4269/ajtmh.19-0119

**Published:** 2020-04-20

**Authors:** Jenna E. Coalson, Ellen M. Santos, Ashley C. Little, Elizabeth J. Anderson, Nancy Stroupe, Maurice Agawo, Mary Hayden, Stephen Munga, Kacey C. Ernst

**Affiliations:** 1 Center for Insect Science, University of Arizona, Tucson, Arizona;; 2 College of Public Health, University of Arizona, Tucson, Arizona;; 3 Kenya Medical Research Institute, Kisumu, Kenya;; 4 Trauma, Health and Hazards Center, University of Colorado Colorado Springs, Colorado Springs, Colorado

## Abstract

Universal “coverage” with long-lasting insecticidal nets (LLINs) is recommended for malaria control in endemic areas, but ownership does not ensure usage. We evaluated relationships between household-level ownership and individual-level usage in western Kenya in 2015. Low-prevalence highland (> 1,500 m) and highly endemic lowland (< 1,200 m) sites were surveyed from July to August 2015. Household members reported long-lasting insecticidal net ownership, use, and barriers to use. Net ownership was categorized as sufficient (≤ 2 people/net), insufficient (> 2 people/net), or none. Each LLIN was assumed to provide access to two people. We surveyed 574 lowland and 643 highland households, with 1,677 and 2,742 members, respectively. More than 98% of lowland households owned LLIN(s); 72.1% owned a sufficient number. Only 37.5% of highland households had sufficient nets. More people used LLINs than were estimated to have access in the lowlands (94.2% versus 85.3%), but proportions were similar in the highlands (54.3% versus 53.3%). Insufficient ownership was most common for larger households in both areas and strongly predicted LLIN usage. In households with insufficient nets, men, school-age children (aged 5–15 years), and nonnuclear family members were less likely to use LLINs; only relationship to the head of the household significantly predicted use in households with sufficient nets. Long-lasting insecticidal nets were widespread in western Kenya in 2015, but insufficient household ownership remained common in the epidemic highlands and in large households. Access seemed to be the primary driver of individual use. To interrupt transmission, LLIN campaigns should improve distribution to large households and promote use among men, school-age children, and nonnuclear family members.

## INTRODUCTION

Malaria is one of the most significant and persistent human infectious diseases, causing an estimated 216 million cases and nearly half a million deaths in 2016.^1^ Extensive global efforts succeeded at reducing the burden of malaria in the first part of this century, but progress has recently stalled. To achieve the goal of 90% malaria reduction by 2030,^2^ we must identify and overcome outstanding challenges to elimination efforts, particularly in sub-Saharan Africa where approximately 90% of malarial deaths are concentrated.^1^ Long-lasting insecticidal nets (LLINs), which can both reduce the abundance of the vector population and provide a physical barrier against feeding/parasite transmission, are a key malaria control technique that have been shown to reduce community prevalence of malaria.^2,3^ Net distribution was initially targeted to groups at high risk of severe malaria—primarily children younger than 5 years and pregnant women. Since 2007, the WHO has recommended universal LLIN coverage to maximize the benefits of vector control and transmission interruption.^4,5^

Three primary measures of LLIN sufficiency are currently recommended to monitor the success of distribution campaigns. The measure with the longest history is ownership of at least one net per household. Ownership of at least one LLIN for every two people in a household, a metric often referred to as “universal coverage,” emerged based on the current WHO recommendation.^5–8^ This recommendation has been in place since 2007, but the reality is that resource limitations still influence distributions in many malaria-endemic areas. Between 2010 and 2017, the proportion of households in sub-Saharan Africa meeting this definition of sufficient LLINs for all occupants doubled to approximately 40%.^1,9^ More recently, household-level recommendations have been supplemented by an individual-level access estimate: the proportion of people with access to an LLIN.^10,11^ This individual-level metric also assumes that each LLIN provides access to two people and better addresses the LLIN availability provided to at least some number of individuals living in households with insufficient LLINs, access which particularly tends to be underestimated in large households that often have some (but not a sufficient number of) LLINs.^11^

However, access to an LLIN in the household does not guarantee use.^12–15^ Detailed household surveys can complement these LLIN metrics to identify factors associated with individual usage, how intra-household distribution is conducted in cases of limited access, and what barriers prevent usage even in the presence of accessible LLINs. Interventions dedicated to net distribution may be insufficient to ensure universal usage of bed nets among people at risk if there are barriers beyond access. Recent research has indicated that patterns of LLIN use in endemic areas still prioritize those at high risk of malaria complications, with adult men and school-age children (5–15 years of age) being less likely to report using LLINs than other groups.^15–18^ A recent analysis of Demographic and Health Survey (DHS) data from 29 countries found that these age and gender differences were attenuated when the households had sufficient nets (at least one for every two people).^15^

Our study aimed to investigate key gaps in household LLIN ownership, the proportion of people with access to an LLIN, and reported LLIN use in two previously studied sites in western Kenya. Transmission of malaria in western Kenya is heterogeneous; prevalence by microscopy ranges from approximately 3.1% in highland epidemic areas to 26.7% in lowland endemic areas despite a history of high LLIN ownership and use.^19^ A combination of mass distributions and routine clinic distributions has been used since 2006 to provide nets to western Kenyan households, with mass distributions occurring more frequently in the lowland endemic area than the epidemic highlands.^20^ The work from 2012 revealed that there were fewer disparities in LLIN use by education and household wealth in the lowlands, where LLINs were more common, suggesting that access may have been the primary driver of disparities in LLIN use.^12^ This analysis also illustrated high variability in access and use patterns within relatively short geographic distances, likely influenced by differential transmission risk. We updated this assessment in 2015, evaluating variations in household LLIN ownership, overall household net ownership in the lowlands compared with the highlands, the number of nets relative to household size, and predictors of individual net use in households with access to nets. We hypothesized that LLIN ownership increased between 2012 and 2015, but that many households in 2015 would continue to own an insufficient number of nets (less than one for every two members), particularly in large households, as recently reported in large, multicountry analyses. In households that owned nets, we hypothesized that there were more likely to be nonusers reported in households with an insufficient number of nets. We further hypothesized school-age children and people who were not part of the nuclear family of the head of household would be less likely to use nets.

## MATERIALS AND METHODS

### Study site.

Villages in the highlands (Kapkangani) and lowlands (Miwani) of western Kenya were selected for inclusion in this study, following a cross-sectional survey performed by our team in these sites in August 2012. Kapkangani is in Nandi district in the western Kenyan highlands, where the altitude ranges from approximately 1,600 to 2,100 m. Malaria transmission in this area is low and unstable with acute seasonal peaks, generally after the rainy season in April–May. The population comprises primarily indigenous Kalenjin people and Luhya settlers from neighboring highland areas. Most people participate in small-scale agriculture, whereas some work as casual laborers on local tea estates. By contrast, Miwani is located on the Kano Plain in western Kenya, 30 km east of Kisumu, where the altitude is approximately 1,200 m above sea level. Malaria transmission in this area is holoendemic and occurs year-round. People are primarily of the Luo tribe and are small-scale farmers with some casual labor on nearby corporate sugarcane and rice farms. This research group had been working in the proposed study sites for approximately 2 years. Few previous studies on malaria had been conducted in these sites. The sites were chosen specifically for their differences in malaria prevalence: low and seasonal in the highlands of Kapkangani versus high year-round in the lowlands of Miwani.

### Study personnel training.

A 4-day classroom training for all field personnel was conducted to provide an overview of the ethical conduct of research. The curriculum included modules on recruitment, informed consent, confidentiality, survey administration, and quality control for data collection. Two weeks of field training was conducted following classroom sessions to implement the data collection tools, identify gaps in the process, and give teams an opportunity to receive guidance from the principal investigator and other senior personnel before implementation.

### Ethics statement.

This study was approved by the Kenya Medical Research Institute (KEMRI) as minimal risk under protocol SSC 2810. The University of Arizona Institutional Review Board deferred review of this study to KEMRI. Permission was sought from local leaders and community members through individual and community meetings before initiation of the study. Individuals aged 18 years and older provided informed consent for the study. Youths aged 7–17 years were asked for their signature or mark of assent for malaria testing.

### Study site census enumeration.

A full census of the population was undertaken in 2013 and updated in July 2014 for five sublocations, two in the lowland area of Miwani and three in the highland area of Kapkangani. Household members were listed by age, gender, and relationship to household head. Latitude, longitude, and elevation were recorded using handheld Garmin global positioning system units.

### Study design and sampling.

The present analysis involved a cross-sectional survey implemented shortly after the onset of the rainy season in 2015, with data collection occurring between June and August. Household sampling was linked to a previous 12-month cohort study in the study site.

The cohort study involved 250 households, 50 randomly selected from each of the five sublocations from the census list enumerated in 2013. To sample additional households for the cross-sectional survey, a list was created of all households from the updated 2014 census that fell within a 1-km buffer of any cohort house in ArcGIS (Esri, Redlands, CA). In the more densely populated lowlands, this included nearly all households in the study area. Additional households were sampled proportional to size for each village in the highlands and in the lowlands. Randomly ordered lists were generated for each village that included an oversample of households by 20% to supplement village sample sizes in case household members were all absent or declined to participate. Households were recruited until the desired sample size for each village was obtained.

### Recruitment and data collection.

Male or female heads of households were approached in person by the field team staff for recruitment. A household was defined as individuals who regularly eat meals together. Households were included if they had resided in the study area for a period of at least 1 month. Households of all sizes and configurations were included.

The primary female caretaker was interviewed to list all household members and bed nets and complete associated household-level, individual-level, and bed net–level survey forms. In the absence of a female caretaker, the male head of the household was surveyed to obtain information. The surveys collected data about the socioeconomic status, bed net ownership, perceptions of malaria risk, barriers to use of various malaria control strategies, and net care/misuse. Individual data were collected on demographics, net use, and recent malaria illness. The bed net forms collected data on net age, quality, and use on the previous night.

### Data management and analysis.

All field teams had a team leader who reviewed and initialed all forms before putting them in envelopes sorted by household. On returning the forms to the research office, a second level of quality control was conducted by the site lead. If discrepancies were identified, the forms were returned to the field team for correction the following day.

Socioeconomic status of the households was estimated using an asset index, with contributing items weighted based on the inverse frequency of their ownership in the community. These index scores were then grouped into quartiles to estimate relative wealth across the entire study population, defined at the household level. A household’s perceived malaria risk was calculated as a composite score from two questions which asked the respondent to rank the seriousness of malaria as a problem: 1) for the family and 2) for the community on scales of 1–5, with 1 representing “not at all serious” and 5 representing “extremely serious.” The sum of the two scores was then grouped as “low” (sum of 2–4), “moderate” (sum of 5–7), or “high” (sum of 8–10).

Analysis was performed with Statistical Analysis Systems version 9.4 (SAS Institute, Cary, NC) to assess gaps in LLIN access and use. Several common LLIN indicators were calculated, most predicated on the assumption that all household members have access to a net if there is at least one available for every two household members.^10,15,21–23^

1. Proportion of households with at least one LLIN (household level)
2. Proportion of households with at least one LLIN for every two people (household level): that is, sufficient LLIN ownership to achieve the WHO recommendation for “universal coverage”
3. Proportion of population with access to an LLIN in their household (individual level): the proportion of each household with access to a net was calculated by multiplying the number of LLINs owned by two and dividing that by the number of reported household members, capped at the maximum coverage of 1.0. This value was assigned to all individuals in the household, and population means of these values were calculated.
4. Proportion of population that reported sleeping under an LLIN the previous night (individual level): the number of individuals reporting the use of an LLIN on the previous night divided by the number for whom the behavior was recorded.
5. Proportion of existing LLINs used the previous night (net level)

We assessed distributions and predictors for gaps in both access to nets at a household level and for household- and individual-level choices to use the LLINs at different levels of household access (ratio of nets to household members). Most outcomes were analyzed dichotomously: households with versus without any LLINs, households with sufficient versus insufficient access among those with at least one net, and individuals who reported using versus not using LLINs. We also performed an exploratory qualitative assessment of households underusing nets (i.e., those where fewer people used nets than had access, assuming two people per net) and households that had unused LLINs.

All potential predictors were categorical, except the total number of household members, which was evaluated both as a linear variable and categorically to allow for nonlinearity. Wilcoxon–Mann–Whitney and chi-squared tests of association were used for the continuous predictors and categorical predictors in univariate analyses, respectively. Fisher’s exact test was used when cells had expected values less than 5. Logistic regression was also used to evaluate the strength of each relationship. Given a priori knowledge about the major differences in demographics and malaria risks between the highlands and lowlands, all models were stratified by site.

## RESULTS

### Population characteristics.

The survey included 643 households in the highlands and 574 households from the lowlands, comprising 2,742 and 1,677 people, respectively. Households from the highlands and lowlands had significantly different distributions of all demographic, socioeconomic, and net-related variables (Table 1).

**Table 1:** Household and individual characteristics, western Kenya, cross-sectional study, 2015

	Highland sites, *n* (%)	Lowland sites, *n* (%)	*P*-value
Number of households	643 (52.8%)	574 (47.2%)	
Household numbers by sublocation	Chepsonoi: 222 (34.5%)	Kabar central: 316 (55.1%)	
	Kiborgok: 204 (31.7%)	Kabar west: 258 (45.0%)	
	Tindinyo: 217 (33.8%)		
Number of people	2,742 (62.1%)	1,677 (37.9%)	
People by sublocations	Chepsonoi: 835 (30.5%)	Kabar central: 843 (50.3%)	
	Kiborgok: 1,007 (36.7%)	Kabar west: 834 (49.7%)	
	Tindinyo: 900 (32.8%)		
Household composition			
Number of household members, median (range)	4 (1–12)	3 (1–9)	< 0.0001
1–2	161 (25.0%)	276 (48.1%)	
3–4	199 (31.0%)	190 (33.1%)	
5–6	177 (27.5%)	85 (14.8%)	
> 6	106 (16.5%)	23 (4.0%)	
Child younger than 5 years living in house			< 0.0001
No	367 (57.1%)	395 (68.8%)	
Yes	276 (42.9%)	179 (31.2%)	
School-aged child (5–15 years) living in house			< 0.0001
No	233 (36.2%)	311 (54.2%)	
Yes	410 (63.8%)	263 (45.8%)	
Perceived malaria severity*			< 0.0001
Low	179 (28.4%)	75 (13.1%)	
Moderate	388 (61.5%)	211 (36.9%)	
High	64 (10.1%)	286 (50.0%)	
Socioeconomic status			
Asset ownership quartile			< 0.0001
Low	165 (25.8%)	123 (21.4%)	
Second	217 (33.9%)	100 (17.4%)	
Third	128 (20.0%)	160 (27.9%)	
High	130 (20.3%)	191 (33.3%)	
Education of the female head of household			0.0023
None or some primary	316 (49.8%)	222 (39.2%)	
Completed primary but not secondary	218 (34.3%)	247 (43.6%)	
Completed secondary	50 (7.9%)	47 (8.3%)	
No female head or other education	51 (8.0%)	51 (9.0%)	
Main building quality†			< 0.0001
Low	52 (8.1%)	200 (35.7%)	
Moderate	412 (64.5%)	294 (52.4%)	
High	175 (27.4%)	67 (11.9%)	
Bed net data			< 0.0001
Number of nets owned, median (range)	1 (0–7)	1 (0–5)	
Last reported bed net distribution			
< 1 year ago	258 (40.4%)	372 (65.6%)	
1–3 years ago	352 (55.2%)	179 (31.6%)	
4–5 years ago	12 (1.9%)	2 (0.4%)	
> 5 years ago	3 (0.5%)	14 (2.5%)	
Unsure	13 (2.0%)	0 (0.0%)	
Bed net received in last distribution			< 0.0001
No	216 (34.0%)	25 (4.4%)	
Yes	418 (65.7%)	541 (95.6%)	
Unsure	2 (0.3%)	0 (0.0%)	
Household net ownership			< 0.0001
None	203 (31.6%)	11 (1.9%)	
Insufficient (< 1 for every two people)	199 (31.0%)	149 (26.0%)	
Sufficient (1+ for every two people)	241 (37.5%)	414 (72.1%)	
Household member net usage			< 0.0001
No members use nets	206 (32.1%)	15 (2.6%)	
Some members use nets	158 (24.7%)	38 (6.6%)	
All members use nets	277 (43.2%)	519 (90.7%)	
Individual characteristics			
Age category (years)			0.01
< 5	366 (15.6%)	233 (15.7%)	
5–15	895 (38.2%)	499 (33.7%)	
> 15–30	420 (17.9%)	343 (23.1%)	
> 30–60	506 (21.6%)	308 (20.8%)	
> 60	155 (6.6%)	100 (6.7%)	
Gender			0.67
Female	1,527 (55.8%)	925 (55.2%)	
Male	1,209 (44.2%)	752 (44.8%)	
Relationship to head of household			< 0.0001
Self/spouse	726 (31.0%)	601 (40.3%)	
Son/daughter	1,096 (46.8%)	654 (43.9%)	
Aunt/uncle	25 (1.1%)	21 (1.4%)	
Niece/nephew	105 (4.5%)	16 (1.1%)	
Grandparent	91 (3.9%)	12 (0.8%)	
Other	300 (12.8%)	186 (12.5%)	
Sleep structure used			
Main house	2056 (87.8%)	1,354 (90.9%)	
Store	1 (0.0%)	3 (0.2%)	
Kitchen	169 (7.2%)	110 (7.4%)	
Grandparent’s house	30 (1.3%)	5 (0.3%)	
Other	87 (3.7%)	18 (1.2%)	
Household net ownership level			< 0.0001
No nets	831 (30.3%)	23 (1.4%)	
Insufficient (< 1 for every two people)	1,140 (41.6%)	685 (40.8%)	
Sufficient (1+ for every two people)	771 (28.1%)	969 (57.8%)	
Used a net last night			< 0.0001
No	1,070 (45.7%)	87 (5.8%)	
Yes	1,273 (54.3%)	1,404 (94.2%)	
Any problem hanging a net over their sleep space?			< 0.01
No	2,242 (95.8%)	1,390 (93.7%)	
Yes	98 (4.2%)	94 (6.3%)	
What are the problems faced when hanging a net over their sleep space?			
No place to hang	34 (36.6%)	25 (29.8%)	
Net too short	1 (1.1%)	0 (0.0%)	
No bed	36 (38.7%)	7 (8.3%)	
Net too small	1 (1.1%)	7 (8.3%)	
Too hot	13 (14.0%)	2 (2.4%)	
Too dirty	0 (0.0%)	1 (1.2%)	
Not enough nets	1 (1.1%)	31 (36.9%)	
Other	7 (7.5%)	11 (13.1%)	
Diagnosed with malaria in the past year?			0.02
No	984 (42.1%)	570 (38.4%)	
Yes	1,355 (57.9%)	915 (61.6%)	
When was the last malaria diagnosis?			< 0.0001
Within last month	336 (24.9%)	360 (39.3%)	
1–3 months ago	414 (30.6%)	349 (38.1%)	
4–6 months ago	339 (25.1%)	106 (11.6%)	
7 months–1 year ago	263 (19.5%)	100 (10.9%)	

* The respondent to the household questionnaire (typically the female head of the household) was asked to rank malaria as a problem both for their family and for their community on a scale of 1–5 (with 1 being “not at all serious” and 5 being “extremely serious”). The sum of the two values was then categorized as “low” (sum 2–4), “moderate” (sum 5–7), or “high” (sum 8–10) perceived severity.

† Building quality was based on an index variable calculated from the total number of finished materials used for the walls, floor, and roof of the structure (0, 1, 2, or 3), with an adjustment based on the field-worker’s rating of household quality on a scale of 1–5. Households receiving the median value of the index score (2.5) were defined as “moderate” quality, whereas those below and above were respectively defined as “low” or “high” quality.

Men were underrepresented in the study. Approximately 72.5% of respondents for the household form were female. The household listings included more women (55.6%) than men (44.4%) in both the highlands and lowlands, and nearly half of the participants (47.9%) were adults aged > 15 years. Of the 4,419 reported household members, 3,834 (86.8%) were present and filled out individual forms reporting on bed net usage. Men were disproportionately likely to be absent at the time of the survey, with data on their net use missing; they accounted for 64.1% of the people who were missing net use data.

### Progress on net indicators.

Distributions of LLIN indicators and characteristics by site (highlands versus lowlands) and sublocations from the 2015 survey are provided in Table 2. There was evidence of considerable improvement on all LLIN indicators in 2015 compared with a similar cross-sectional survey our team undertook in these same sites in August 2012,^12^ although the reported discrepancies between the highlands and lowlands persisted. We found that the spatial heterogeneity was pronounced even between sublocations within the highlands and lowlands in 2015, with better LLIN indicators in Chepsonoi villages than Kiborgok or Tindinyo in the highlands.

**Table 2:** Bed net indicators by sublocation, western Kenya, cross-sectional study, 2015

	Highland sites				Lowland sites		
Sublocation	Chepsonoi	Kiborgok	Tindinyo	Total	Kabar central	Kabar west	Total
Sample sizes, *n*							
Number of households	222	204	217	643	316	258	574
Number of people	835	1,007	900	2,742	843	834	1,677
Number of nets	351	275	248	874	463	421	884
LLIN indicators (%)							
Percentage of households with at least one LLIN	81.5	62.8	60.4	68.4	100	95.7	98.1
Percentage of households with at least one LLIN for every two people	53.2	27.9	30.4	37.5	75.6	67.8	72.1
Proportion of population with access to an LLIN in their household*	65.9	48.7	46.8	53.3	87.9	82.7	85.3
Proportion of the population that slept under an LLIN on the previous night	70.8	46.6	47.4	54.3	98.3	89.4	94.2
Proportion of existing LLINs used on the previous night	98.6	100	98.8	99.1	99.6	96.6	98.2
Number of people using each LLIN on the previous night (of LLINs in use), mean (range)	1.61 (1–5)	1.67 (1–3)	1.76 (1–4)	1.67 (1–5)	1.84 (1–5)	1.84 (1–5)	1.84 (1–5)
Net sources (%)							
Mass distribution	87.7	75.9	90.2	84.7	88.1	91.0	89.5
Clinic	4.9	23.0	8.1	11.5	2.9	3.9	3.4
Market/shop/supermarket	4.0	0.4	1.2	2.1	1.3	1.0	1.2
Village elders/community member/relative	3.1	0.4	0	1.5	5.5	2.7	4.2
Other/do not know	0.3	0.4	0	0.2	2.2	1.5	1.9
Net age (years) (%)							
≤ 1	34.6	46.0	52.5	43.2	10.6	31.4	20.5
> 1–2	54.0	3.3	28.1	30.7	72.0	53.0	63.0
> 2–3	10.3	25.9	1.6	12.8	9.9	0.7	5.6
> 3	0.9	24.4	17.9	13.1	7.5	11.9	9.6
Do not know/decline	0.3	0.4	0	0.2	0	2.9	1.4
Net condition (%)†							
Excellent (no major holes)	50.3	65.2	68.7	60.2	89.2	46.2	68.8
Good (1–5 medium or large holes)	36.6	18.3	21.1	26.5	8.2	30.4	18.8
Fair (many medium holes and < 5 large holes)	12.0	12.1	9.4	11.3	2.4	18.0	9.8
Poor (many medium and large holes)	1.1	4.4	0.8	2.1	0.2	5.4	2.7

LLIN = long-lasting insecticidal net.

* “Proportion of the population with access to an LLIN” was calculated using the assumption that each LLIN a household owned provided access to up to two household members, as recommended by the Roll Back Malaria Measurement and Evaluation Reference Group.^10^

† Net condition was evaluated by the study team and guided by comparison to images.

Household ownership of at least 1 bed net essentially doubled from 2012 to 2015 in both the highlands (from 37% to 68%) and lowlands (from 53% to 98%), seemingly attributable to mass distribution campaigns. In 2012, only 8% of nets in the highlands and 36% in the lowlands were reportedly obtained from mass distributions (free distribution at the clinic was the primary source).^12^ Only 3 years later, the vast majority (85% and 90%, respectively) were reported to have come from mass distributions. Progress on the proportion of households owning sufficient nets for a ratio of 1:2 people was even more striking from 2012 to 2015, increasing from only 5% to 37.5% in the highlands and 10% to 72% in the lowlands, but there remained a significant gap on this indicator. In the lowlands, where the prevalence of malaria is higher, an LLIN distribution had taken place in September 2014, 8 months before the survey, aiming to provide at least one net for every two individuals enumerated in the households without a per household cap/ceiling. Although 32% of households in the highlands did not own any nets in mid-2015, an LLIN distribution effort that coincided with this cross-sectional survey was increasing coverage levels. Of the ∼40% of highland households that reported knowledge of a community distribution campaign within the year of the survey, only 15.9% owned no nets and 28.3% owned insufficient numbers of nets compared with 42.3% and 33.1%, respectively, among the households that were unaware of the recent distribution campaign.

At the individual level, the proportion of the population with access to an LLIN was not calculated in 2012, but the proportion of people who reported using a bed net approximately doubled by 2015, from 22% to 54% in the highlands and from 48% to 94% in the lowlands. In the highlands, this was approximately equal to the estimated 53% of people with access to an LLIN in 2015 (metric not calculated in the 2012 study). In the lowlands, however, a greater proportion of people used nets than the 85% of the population estimated to have access to one. This pattern was reflected in the household use to access ratios: in the lowlands, only 4% of households underused nets (i.e., fewer people used nets than had access, assuming two potential users per net), compared with 21.5% of households in the highlands. In 2012, it was uncommon for nets to go unused, but it was even rarer in 2015 (0.9% of nets in the highlands and 1.8% in the lowlands).

### Predictors of household-level net ownership.

Only 11 households in the lowlands (1.9%) did not own any bed nets in 2015. Eight of these 11 households only had one or two members, and only one of them had a child younger than 5 years living in the house. Eight of the households were in the lowest wealth quartile. Comparatively, in the highlands, nearly a third of households did not own any bed nets in 2015 (31.6%). Households in the lowest wealth quartile were significantly less likely to own any nets than those in higher wealth quartiles, those that could not afford to buy a net were significantly less likely to own a net than those that could, and households with poor construction quality were significantly less likely to own any nets than households with better construction quality. Details are provided in Supplemental Table 1.

**Figure 1.**
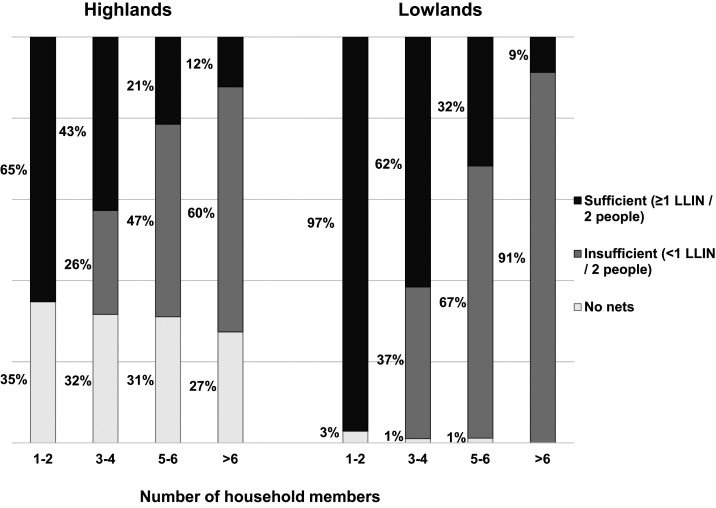
Long-lasting insecticidal net (LLIN) ownership by household size in highland and lowland sites, western Kenya, 2015. Note: It was impossible by definition for households with only one to two members to qualify as having an insufficient number of nets.

Although mass distribution efforts had made great strides since 2012, and most households owned at least one LLIN, many households still had insufficient numbers of nets in both sites, particularly among large households (Figure 1). Of households with six or more members, only 12.3% and 8.7% had sufficient nets in the highlands and lowlands, respectively. Table 3 (highlands) and Table 4 (lowlands) present the distribution of insufficient versus sufficient bed net ownership ratios among households that owned nets and had at least three household members. Households with only one or two members were excluded from tables because ownership of one net inherently qualified the household as having a sufficient number. As indicated in Figure 1, increasing household size was the strongest predictor of having insufficient bed nets in both sites. Households in the lowlands tended to be smaller than those in the highlands, potentially contributing to the discrepancy by site for this indicator.

**Table 3:** Predictors of insufficient long-lasting insecticidal net ownership (< 1 net for every two people) among households that own at least one net, highland sites

		Ownership ratio		ORs predicting insufficient number of nets		
	Total, *n*	Insufficient, *n* (%)	Sufficient, *n* (%)*	Crude OR (95% CI)	Adjusted OR (95% CI) (for hh size)	Adjusted OR (95% CI) (full model)
Households (with 3+ members)†	335	199 (59.4%)	136 (40.6%)			
Household member composition						
Number of household members, mean (range)	5.22 (3–12)	5.73 (3–12)	4.47 (3–11)	**1.62 (1.38–1.90)**		
3–4	136	51 (37.5%)	85 (62.5%)	1.00 (ref)	1.00 (ref)	1.00 (ref)
5–6	122	84 (68.9%)	38 (31.1%)	**3.68 (2.20–6.18)**	**3.68 (2.20–6.18)**	**3.88 (2.18–6.90)**
> 6	77	64 (83.1%)	13 (16.9%)	**8.20 (4.12–16.36)**	**8.20 (4.12–16.36)**	**8.62 (3.88–19.17)**
Child younger than 5 years living in house						
No	154	74 (48.1%)	80 (51.9%)	1.00 (ref)	1.00 (ref)	1.00 (ref)
Yes	181	125 (69.1%)	56 (30.9%)	**2.41 (1.54–3.77)**	**1.65 (1.01–2.68)**	**1.78 (1.05–3.03)**
School-aged child (5–15 years) living in house						
No	73	38 (52.1%)	35 (48.0%)	1.00 (ref)	1.00 (ref)	
Yes	262	161 (61.5%)	101 (38.6%)	1.47 (0.87–2.48)	0.91 (0.51–1.63)	
Perceived malaria severity‡						
Low	91	61 (67.0%)	30 (33.0%)	1.00 (ref)	1.00 (ref)	
Moderate	205	114 (55.6%)	91 (44.4%)	0.62 (0.37–1.03)	**0.54 (0.31–0.95)**	
High	34	22 (64.7%)	12 (35.3%)	0.90 (0.39–2.06)	0.85 (0.35–2.07)	
Socioeconomic status						
Asset ownership quartile						
Low	62	43 (69.4%)	19 (30.6%)	**2.68 (1.34–5.35)**	**4.68 (2.14–10.25)**	**2.54 (1.06–6.05)**
Second	113	73 (64.6%)	40 (35.4%)	**2.16 (1.21–3.86)**	**3.14 (1.63–6.07)**	**2.18 (1.08–4.40)**
Third	77	45 (58.4%)	32 (41.6%)	1.67 (0.89–3.11)	**2.31 (1.14–4.69)**	2.02 (0.94–4.33)
High	83	38 (45.8%)	45 (54.2%)	1.00 (ref)	1.00 (ref)	1.00 (ref)
Education of the female head of household						
None or some primary	172	119 (69.2%)	53 (30.8%)	**5.49 (2.37–12.72)**	**4.21 (1.73–10.29)**	2.60 (0.98–6.90)
Completed primary but not secondary	122	65 (53.3%)	57 (46.7%)	**2.79 (1.19–6.54)**	2.12 (0.86–5.27)	1.31 (0.49–3.52)
Completed secondary	31	9 (29.0%)	22 (71.0%)	1.00 (ref)	1.00 (ref)	1.00 (ref)
No female head or other education	9	5 (55.6%)	4 (44.4%)	3.06 (0.66–14.06)	4.29 (0.86–21.52)	3.53 (0.61–20.32)
Main building quality§						
Low	21	17 (81.0%)	4 (19.0%)	**5.14 (1.61–16.42)**	**7.82 (2.27–26.96)**	**4.83 (1.23–18.96)**
Moderate	216	136 (63.0%)	80 (37.0%)	**2.06 (1.26–3.35)**	**2.59 (1.49–4.47)**	1.77 (0.97–3.25)
High	95	43 (45.3%)	52 (54.7%)	1.00 (ref)	1.00 (ref)	1.00 (ref)

OR = odds ratio. Bolded values were statistically significant at *P* < 0.05.

* Sufficient net ownership was defined as having at least one bed net for every two household members.

† Table excludes 161 households with only one to two members, as these households inherently had sufficient nets for both members by owning one.

‡ The respondent to the household questionnaire (typically the female head of the household) was asked to rank malaria both as a problem for their family and for their community on a scale of 1–5 (with 1 being “not at all serious” and 5 being “extremely serious”). The sum of the two values was then categorized as “low” (sum 2–4), “moderate” (sum 5–7), or “high” (sum 8–10) perceived severity.

§ Building quality was based on an index variable calculated from the total number of finished materials used among the walls, floor, and roof of the structure (0, 1, 2, or 3) with an adjustment based on the field-worker’s rating of household quality on a scale of 1–5. Households receiving the median value of the index score (2.5) were defined as “moderate” quality, whereas those below and above were, respectively, defined as “low” or “high” quality.

**Table 4:** Predictors of insufficient long-lasting insecticidal net ownership (< 1 net for every two people) among households that own at least one net, lowland sites

		Ownership ratio		ORs predicting insufficient number of nets		
	Total, *n*	Insufficient, *n* (%)	Sufficient, *n* (%)*	Crude OR (95% CI)	Adjusted OR (95% CI) (for hh size)	Adjusted OR (95% CI) (full model)
Households with 3+ members†	295	149 (50.5%)	146 (49.5%)			
Household member composition						
Number of household members, mean (range)	4.29 (3–9)	4.60 (3–9)	3.97 (3–7)	**1.48 (1.22–1.78)**		
3–4	188	71 (37.8%)	117 (62.2%)	1.00 (ref)	1.00 (ref)	1.00 (ref)
5–6	84	57 (67.9%)	27 (32.1%)	**3.68 (2.20–6.18)**	**3.68 (2.20–6.18)**	**3.42 (1.93–6.07)**
> 6	23	21 (91.3%)	2 (8.7%)	**8.20 (4.12–16.36)**	**8.20 (4.12–16.36)**	**14.47 (3.19–65.74)**
Child younger than 5 years living in house						
No	130	45 (34.6%)	85 (65.4%)	1.00 (ref)	1.00 (ref)	1.00 (ref)
Yes	165	104 (63.0%)	61 (37.0%)	**3.22 (1.99–5.20)**	**2.65 (1.60–4.40)**	**2.89 (1.71–4.89)**
School-aged child (5–15 years) living in house						
No	77	37 (48.1%)	40 (52.0%)	1.00 (ref)	1.00 (ref)	
Yes	218	112 (51.4%)	106 (48.6%)	1.14 (0.68–1.92)	0.67 (0.38–1.19)	
Perceived malaria severity‡						
Low	37	15 (40.5%)	22 (59.5%)	1.00 (ref)	1.00 (ref)	
Moderate	111	66 (59.5%)	45 (40.5%)	**2.15 (1.01–4.59)**	1.95 (0.87–4.36)	
High	145	67 (46.2%)	78 (53.8%)	1.26 (0.61–2.62)	1.19 (0.54–2.60)	
Socioeconomic status						
Asset ownership quartile						
Low	50	27 (54.0%)	23 (46.0%)	1.26 (0.65–2.47)	1.12 (0.55–2.30)	
Second	51	25 (49.0%)	26 (51.0%)	1.03 (0.53–2.01)	1.07 (0.53–2.16)	
Third	84	44 (52.4%)	40 (47.6%)	1.18 (0.67–2.09)	1.09 (0.59–2.00)	
High	110	53 (48.2%)	57 (51.8%)	1.00 (ref)	1.00 (ref)	
Education of the female head of household						
None or some primary	118	52 (44.1%)	66 (55.9%)	1.05 (0.46–2.41)	0.99 (0.41–2.43)	
Completed primary but not secondary	143	81 (56.6%)	62 (43.4%)	1.74 (0.77–3.95)	1.73 (0.72–4.16)	
Completed secondary	28	12 (42.9%)	16 (57.1%)	1.00 (ref)	1.00 (ref)	
No female head or other education	2	1 (50.0%)	1 (50.0%)	1.33 (0.08–23.54)	2.18 (0.12–39.26)	
Main building quality§						
Low	112	65 (58.0%)	47 (42.0%)	**2.21 (1.05–4.67)**	**2.65 (1.18–5.97)**	**2.82 (1.22–6.51)**
Moderate	137	65 (47.4%)	72 (52.6%)	1.44 (0.70–2.99)	1.54 (0.70–3.39)	1.69 (0.75–3.84)
High	39	15 (38.5%)	24 (61.5%)	1.00 (ref)	1.00 (ref)	1.00 (ref)

OR = odds ratio. Bolded values were statistically significant at *P* < 0.05.

* Sufficient net ownership was defined as having at least one bed net for every two household members.

† Table excludes 268 households with only one to two members, as these households inherently had sufficient net ownership by owning one.

‡ The respondent to the household questionnaire (typically the female head of household) was asked to rank malaria both as a problem for their family and for their community on a scale of 1–5 (with 1 being “not at all serious” and 5 being “extremely serious”). The sum of the two values was then categorized as “low” (sum 2–4), “moderate” (sum 5–7), or “high” (sum 8–10) perceived severity.

§ Building quality was based on an index variable calculated from the total number of finished materials used among the walls, floor, and roof of the structure (0, 1, 2, or 3), with an adjustment based on the field-worker’s rating of household quality on a scale of 1–5. Households receiving the median value of the index score (2.5) were defined as “moderate” quality, whereas those below and above were respectively defined as “low” or “high” quality.

Given that previous bed net distribution tended to target pregnant women and children younger than 5 years, we hypothesized that households in which there was at least one child younger than 5 years would be more likely to have sufficient nets, but this was not the case. Even after adjusting for household size, households with a child younger than 5 years had 1.65 times the odds (95% CI: 1.01–2.68) of having an insufficient number of bed nets than households that had no children younger than 5 years in the highlands, and 2.65 times the odds (95% CI: 1.60–4.40) in the lowlands. As expected based on previous data^12^ and the results from the net ownership data presented earlier, low socioeconomic status was associated with insufficient net ownership in the highlands; however, there was weak evidence of a relationship between net ownership and socioeconomic status variables in the lowlands.

Given the concurrent mass distribution campaign in the highlands, we ran a sensitivity analysis on the predictors of insufficient net ownership. Despite a loss of precision due to the smaller sample sizes, there were no notable differences in predictor patterns among the ∼40% of households in which the interviewee was aware of the recent community distribution campaign compared with those that were not.

### Predictors of individual bed net usage.

**Figure 2.**
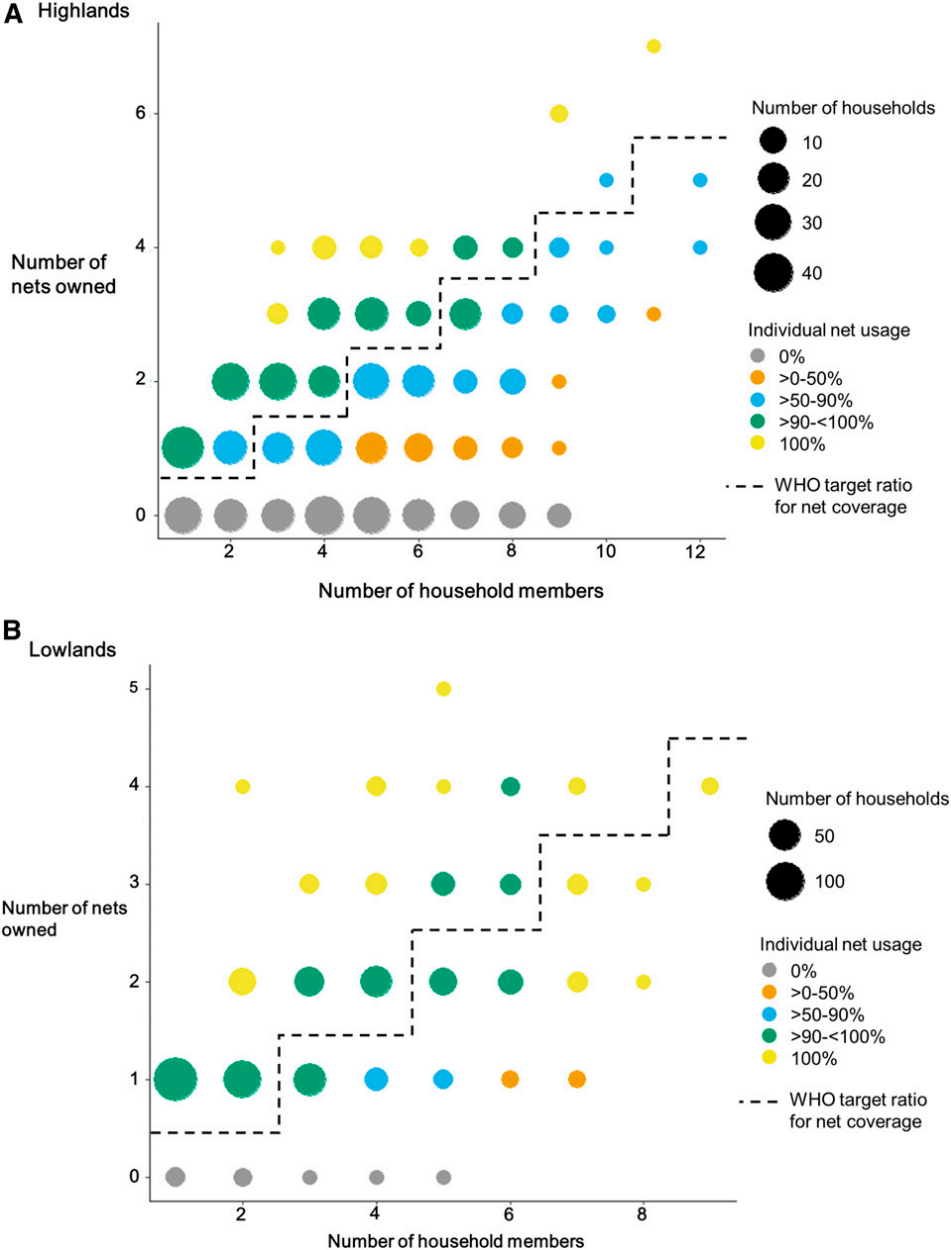
Long-lasting insecticidal net use and ratio of household size to the number of nets owned in **(A)** the highlands and **(B)** the lowlands of western Kenya.

Figure 2 displays the relationship between number of nets owned, number of household members, and individual-level net usage for the highlands (Panel A) and the lowlands (Panel B), with the dotted line dividing households with access to at least one net for every two members (sufficient nets). In the highlands, the percentage of individuals who used nets was highest for the households with sufficient nets, and usage was poorer in households with insufficient nets. Alternatively, in the lowlands, the reported personal net use was high even in households where access was considered insufficient, with > 90% of people sleeping under bed nets in all households that owned at least one net, regardless of the number of members.

Predictors of individual-level net use were evaluated among people in households that owned at least one net, where nonuse of a net may reflect either individual preference or household prioritization of limited nets among members (Table 5). Nearly all (1,404 of 1,469 [95.6%]) lowland residents in houses with at least one net reported using a bed net on the previous night, compared with only 78.1% (1,273 of 1,630) in the highlands. Sufficient household-level net ownership was significantly associated with members using nets in both sites, although the relationship was stronger in the highlands than the lowlands, corresponding to observations from Figure 2. In the highlands, 95.1% of people used a net in households with sufficient nets, compared with only 66.2% of people in households with insufficient net ownership (odds ratio [OR] = 9.86, 95% CI: 6.78–14.35). In the lowlands, the corresponding numbers were 98.4% and 91.6% (OR = 5.56, 95% CI: 3.05–10.13). Furthermore, although few people reported having problems hanging a net over their sleep space overall in either site, those in the lowlands who reported having a problem were much less likely to have used a net (OR = 0.10, 95% CI: 0.05–0.18) than those who did not report problems, and many stated that the problem was “not having enough nets.” In the highlands, the opposite relationship was observed. People who reported having problems hanging a net were more likely to have used a net than those who did not report any problems (OR = 2.17, 95% CI: 1.15–4.13). Only one person said that “not having enough nets” was a problem; the most common problems reported were not having a bed or a place to hang the net.

**Table 5:** Predictors of net use by individuals in households that owned at least one net

	Highlands			Lowlands		
	Total, *n*	Used a net last night, *n* (%)	OR (95% CI)	Total, *n*	Used a net last night, *n* (%)	OR (95% CI)
Total number of people	1,630	1,273 (78.1%)		1,469	1,404 (95.6%)	
Demographics						
Age category (years)						
< 5	250	219 (87.6%)	1.00 (ref)	232	226 (97.4%)	1.00 (ref)
5–15	625	408 (65.3%)	**0.27 (0.18–0.40)**	491	451 (91.9%)	**0.30 (0.13–0.72)**
> 15–30	286	216 (75.5%)	**0.44 (0.28–0.69)**	337	325 (96.4%)	0.72 (0.27–1.94)
> 30–60	362	332 (91.7%)	1.57 (0.92–2.66)	305	299 (98.0%)	1.32 (0.42–4.16)
> 60	106	98 (92.5%)	1.73 (0.77–3.91)	96	96 (100.0%)	∞*
Gender						
Female	971	805 (82.9%)	**1.98 (1.56–2.51)**	870	847 (97.4%)	**2.78 (1.65–4.67)**
Male	656	466 (71.0%)	1.00 (ref)	599	557 (93.0%)	1.00 (ref)
Relationship to head of household						
Nuclear family (self/spouse and child)	1,277	1,055 (82.6%)	**4.75 (2.89–7.81)**	1,236	1,201 (97.2%)	**4.16 (1.40–12.38)**
Aunt/uncle/niece/nephew	68	34 (50.0%)	1.00 (ref)	37	33 (89.2%)	1.00 (ref)
Other	285	184 (64.6%)	**1.82 (1.07–3.11)**	195	170 (87.2%)	0.82 (0.27–2.52)
Sleep structure used						
Main house	1,422	1,159 (81.5%)	**3.63 (2.68–4.93)**	1,332	54 (95.9%)	1.88 (0.93–3.78)
Other	208	114 (54.8%)	1.00 (ref)	136	126 (92.6%)	1.00 (ref)
Bed net use						
Household net access†						
Insufficient number of nets	959	635 (66.2%)	1.00 (ref)	607	556 (91.6%)	1.00 (ref)
Sufficient number of nets	671	638 (95.1%)	**9.86 (6.78–14.35)**	862	848 (98.4%)	**5.56 (3.05–10.13)**
Any problem hanging a net over their sleep space?						
No	1,533	1,190 (77.6%)	1.00 (ref)	1,382	1,339 (96.9%)	1.0 (ref)
Yes	94	83 (88.3%)	**2.17 (1.15–4.13)**	81	61 (75.3%)	**0.10 (0.05–0.18)**
What are the problems faced when hanging a net over their sleep space?						
No place to hang	32	29 (90.6%)		24	24 (100.0%)	
Net too short	1	0 (0.0%)		0	–	
No bed	36	36 (100.0%)		7	7 (100.0%)	
Net too small	1	1 (100.0%)		7	7 (100.0%)	
Too hot	12	8 (66.7%)		2	2 (100.0%)	
Too dirty	0	–		1	0 (0.0%)	
Not enough nets	1	0 (0.0%)		19	2 (10.5%)	
Other	6	3 (50.0%)		11	11 (100.0)	
Malaria variables						
Diagnosed with malaria in past year?						
No	647	486 (75.1%)	1.00 (ref)	566	541 (95.6%)	1.00 (ref)
Yes	982	786 (80.0%)	**1.33 (1.05–1.68)**	897	858 (95.7%)	1.02 (0.61–1.70)
When was the last malaria diagnosis?						
Within last month	267	228 (85.4%)	**2.21 (1.36–3.58)**	350	330 (94.3%)	0.17 (0.02–1.27)
1–3 months ago	322	262 (81.4%)	**1.65 (1.06–2.57)**	342	324 (94.7%)	0.18 (0.02–1.39)
4–6 months ago	226	174 (77.0%)	1.27 (0.80–2.01)	106	105 (99.1%)	1.07 (0.07–17.37)
7 months–1 year ago	164	119 (72.6%)	1.00 (ref)	99	98 (99.0%)	1.00 (ref)
Household perception of malaria risk‡						
Low	449	320 (71.3%)	1.00 (ref)	166	155 (93.4%)	1.00 (ref)
Moderate	983	804 (81.8%)	**1.71 (1.40–2.35)**	563	526 (93.4%)	1.01 (0.50–2.02)
High	173	130 (75.1%)	1.22 (0.82–1.82)	734	717 (97.7%)	**2.99 (1.38–6.52)**

OR = odds ratio. Bolded values were statistically significant at *P* < 0.05.

* CI not calculated as all individuals in the age category used a net on the previous night in the lowlands.

† Sufficient household net access was defined as having at least one bed net for every two household members. Insufficient access was defined as having at least one net but less than one for every two household members.

‡ The respondent to the household questionnaire (typically the female head of household) was asked to rank malaria both as a problem for their family and for their community on a scale of 1–5 (with 1 being “not at all serious” and 5 being “extremely serious”). The sum of the two values was then categorized as “low” (sum 2–4), “moderate” (sum 5–7), or “high” (sum 8–10) perceived severity.

Variables related to malaria risk were also associated with reported net use (Table 5). Although perceived severity of malaria was assessed at the household and not at the individual level, people in the lowlands who lived in households with high perceived risk were significantly more likely to use a net than those in households reporting low or moderate perceived risk. The relationship was inconsistent in the highlands, where households generally reported lower perceived risk than in the lowlands. Individual perceptions of risk may be influenced by the timing of last malaria diagnosis. People in the highlands were most likely to report using a net if they had been diagnosed with malaria in the past month, with use becoming increasingly less prevalent among people whose last malaria diagnosis was older. However, there was no significant association between the timing of last malaria diagnosis and net use in the lowlands.

**Figure 3.**
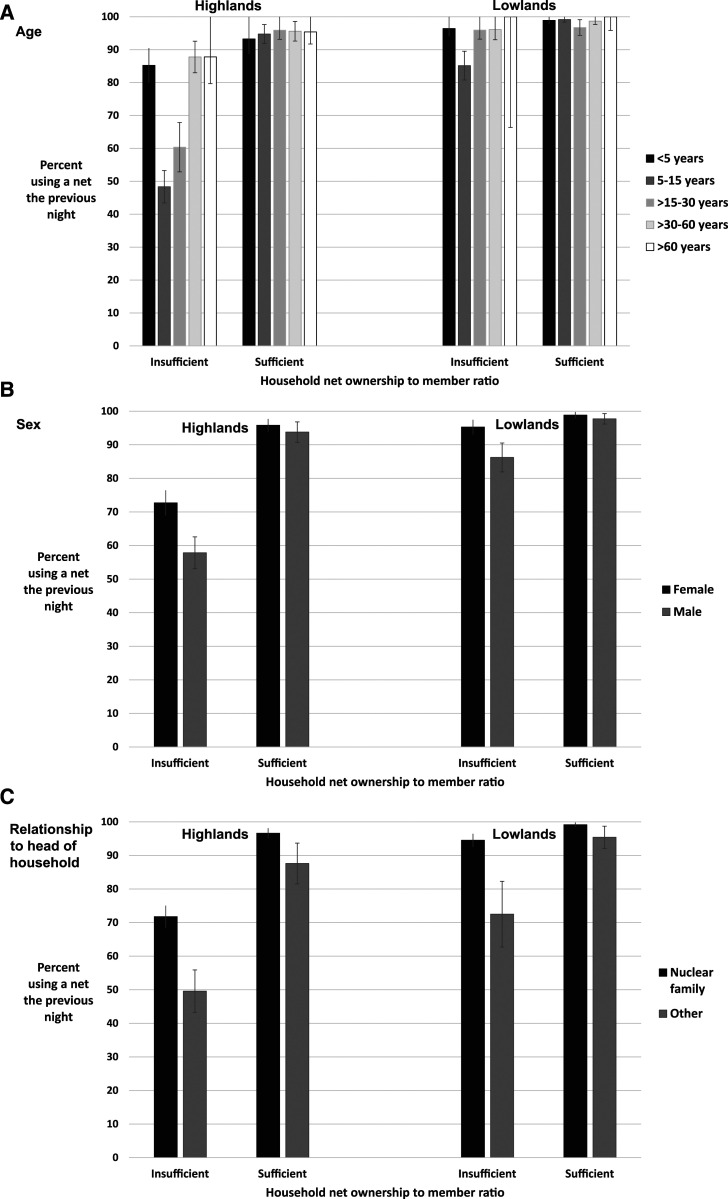
Use of long-lasting insecticidal nets by (**A**) age, (**B**) gender, and (**C**) relationship to the head of the household, stratified by site and household LLIN ownership ratio. Note: Bars represent the 95% CI of each estimate using Wald for those with counts > 5 and the Clopper–Pearson exact method for those with counts ≤ 5.

Finally, individual demographic characteristics were also associated with reported net use. People who belonged to the nuclear family of the head of the household were significantly more likely to report using a net than secondary family members (aunts, uncles, nieces, and nephews) in both sites. Significantly fewer men reported sleeping under a net than women in all age categories. School-aged children (aged 5–15 years) made up more than 60% of all nonusers of nets and had roughly 0.3 times the odds of using a net as children younger than 5 years in both sites. Young adults (aged > 15–30 years) were also less likely to use nets than children younger than 5 years, particularly in the highlands. Notably, the number of nets owned by the household (i.e., access) modified the relationships between net use and age and gender (Figure 3, Table 6). The differences in net use by age or gender were not statistically significant in households that owned a sufficient number of nets in either the lowlands or highlands. However, relationship to the head of the household remained strongly predictive of net use in both households with insufficient and sufficient nets, with people who were not members of the nuclear family being significantly less likely to use nets in both settings.

**Table 6:** Individual-level predictors of net use stratified by site and household net ownership*

	Highlands		Lowlands	
	OR (95% CI) for using a net		OR (95% CI) for using a net	
Household net ownership*	Insufficient	Sufficient	Insufficient	Sufficient
Age category (years)				
< 5	1.00 (ref)	1.00 (ref)	1.00 (ref)	1.00 (ref)
5–15	**0.16 (0.10–0.26)**	1.30 (0.44–3.83)	**0.21 (0.08–0.55)**	1.28 (0.12–14.29)
> 15–30	**0.26 (0.16–0.44)**	1.70 (0.47–6.07)	0.87 (0.25–3.09)	0.33 (0.04–2.68)
> 30–60	1.25 (0.68–2.29)	1.58 (0.50–4.99)	0.91 (0.21–3.93)	0.82 (0.09–8.03)
> 60	1.25 (0.45–3.47)	1.50 (0.34–6.53)	∞†	∞†
Gender				
Female	**1.94 (1.48–2.55)**	1.52 (0.75–3.07)	**3.22 (1.76–5.91)**	1.95 (0.67–5.68)
Male	1.00 (ref)	1.00 (ref)	1.00 (ref)	1.00 (ref)
Relationship to head of household				
Nuclear family (self/spouse and child)	**2.58 (1.91–3.49)**	**4.01 (1.95–8.27)**	**6.51 (3.51–12.08)**	**5.66 (1.87–17.08)**
Other	1.00 (ref)	1.00 (ref)	1.00 (ref)	1.00 (ref)

OR = odds ratio. Bolded values were statistically significant at *P* < 0.05.

* Sufficient household net access was defined as having at least one bed net for every two household members. Insufficient access was defined as having at least one net but less than one for every two household members.

† CI not calculated as all individuals in the age category used a net on the previous night in the lowlands.

### Unused and underused nets.

Although it was uncommon for nets to go unused by any household members in the 2012 survey (4.5% of nets in the lowlands and 7.3% in the highlands), essentially all nets were used in the 2015 survey. In 2015, there were only 24 nets in 17 households that had not been used on the previous night—eight of 870 nets in the highlands (0.92%) and 16 of 864 nets in the lowlands (1.85%). Of these 24 nets, 15 (62.5%) belonged to nine households where all members already reported sleeping under other nets on the previous night. Another four nets were reported not to have been used because the typical user had not slept in that space the previous night. No explanation was given by the four households that owned the remaining five nets as to why they had not been used the previous night, despite there being 10 people in the households who reported not having used a net. These five nets represented only 0.3% of all recorded nets in the study sample.

**Figure 4.**
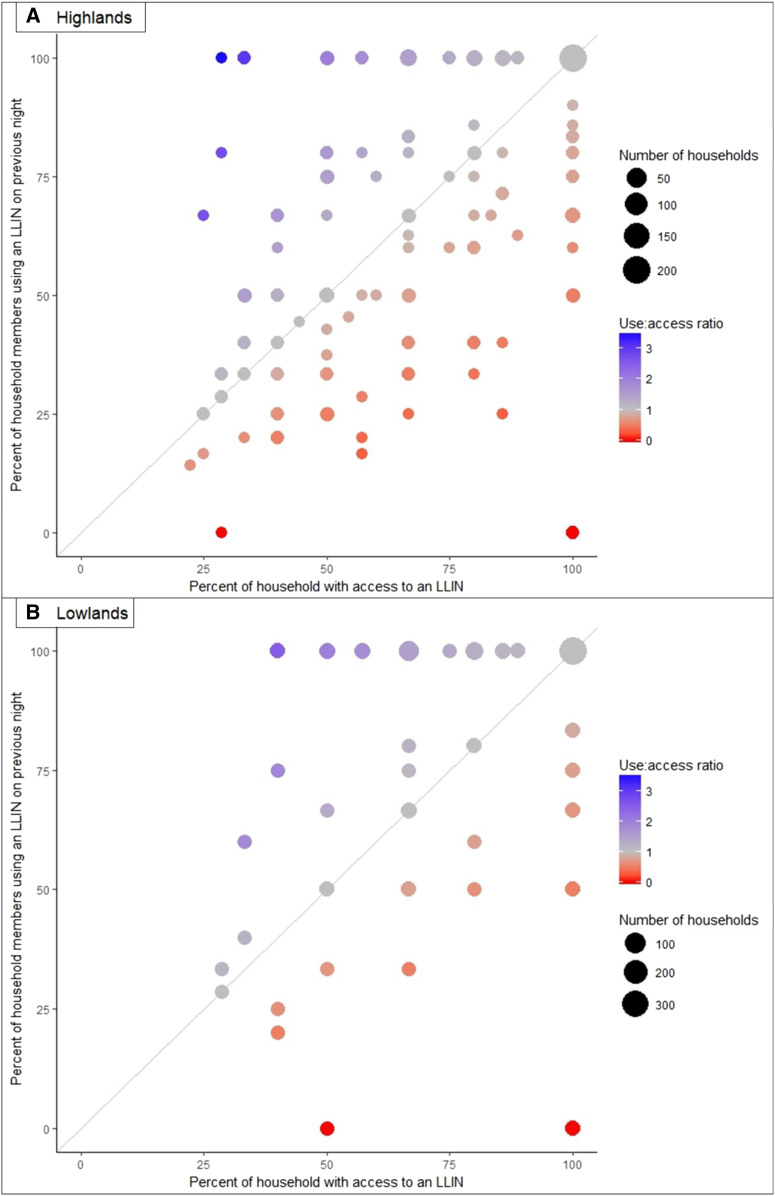
Percent of long-lasting insecticidal net (LLIN) users vs. the percent of the household estimated to have access to an LLIN in the (**A**) highlands and (**B**) lowlands. Note: The percent with access was calculated using all declared household members as the denominator; the percent using a net was calculated among only those members whose net use on the previous night was known.

We further examined the composition of households reporting underused nets, that is, those that reported low net usage relative to their predicted access when assuming two users per net (Figure 4). There were 29 households in the highlands and four in the lowlands that averaged one or fewer users per net, despite there being household members who had not used a net the previous night. The composition was variable, but often consisted of a single head of household living with his/her school-aged or adult children, nonnuclear family members, or both, where either the head of the household (16 households) or a child (three households) used a net alone. There were another seven households in which both the head of the household and a spouse were present and used separate nets, whereas one or more of their school-aged/adult children or nonnuclear family members did not use a net.

## DISCUSSION

Maximizing the protective benefits of LLINs requires both that people have access to LLINs in their homes and that they use them regularly when they have access. This 2015 survey found vast improvements in all indicators of LLIN ownership and use across western Kenyan highland and lowland sites since a similar survey undertaken in the area in 2012,^12^ although gaps remain. Considerable geographic heterogeneity in ownership and use persisted between the highly endemic lowlands and the epidemic highlands, and the 2015 survey found variability on net indicators at even smaller geographic scales sampled within the two larger sites. Nearly all households surveyed in the lowlands owned at least one LLIN and reported individual usage was high; however, in the highlands, ownership was lower, spatially heterogeneous, and associated with socioeconomic status. In all areas, increasing household size was the strongest predictor of households owning an insufficient number of nets to cover all members, assuming two users per net. Several results from the 2015 cross-sectional study suggested that lack of access was the primary limitation to LLIN use in these survey sites: 1) essentially no LLINs went unused unless the household had a surplus; 2) the proportion of people who used a net exceeded the proportion of people estimated to have access if there were two users per net, particularly in the lowlands; 3) owning a sufficient number of nets strongly predicted individual net use compared with households with an insufficient number of nets; and 4) observed overall gender- and age-related discrepancies in net use were minimized or eliminated among households that owned a sufficient number of nets. These results demonstrate the success of LLIN distribution campaigns in western Kenya from 2012 to 2015, but highlight that continuing intervention efforts need to ensure sufficient net distributions to larger households and to minimize gaps at small geographic scales while maintaining the high ownership levels achieved in the most highly burdened regions.

Our results correspond to recently published findings from large-scale multicountry analyses based on DHS and Malaria Indicator Survey (MIS) data. Koenker et al.^11^ reported the same ownership gap in achieving a sufficient number of nets to household members in large households, but emphasized that this ownership indicator underestimates the individual-level access provided by the nets that do exist in “insufficiently” covered households. Individual LLIN usage was roughly equivalent to the “proportion of the population with access to a net” in our 2015 data and, in fact, exceeded it in the lowland area, supporting their conclusion that the population access to LLIN metric is superior to the proportion of households with sufficient nets for evaluating the success of “universal coverage” net distributions.

Our 2015 western Kenya results also supported the age- and gender-specific net use patterns Olapeju et al.^15^ reported from the multicountry DHS/MIS analysis, with disparities that manifested in households with insufficient net ownership but were minimal in households with sufficient nets. This pattern was observed in both the highland and lowland sites in 2015, suggesting that this phenomenon is consistent on large and small scales and not dramatically modified by malaria prevalence. In particular, the finding that school-age children were the least likely age-group to use LLINs has been reported in many sub-Saharan African settings and acknowledged in the most recent Malaria Communication Strategy for Kenya.^15–18,24^ Studies in endemic areas have also suggested that these school-age children have high prevalence of infection and rarely seek treatment.^17,25–29^ If these children are frequent reservoirs of *Plasmodium* infection, relatively low use of LLINs could be making them more frequent targets for mosquito blood meals and enabling considerable contributions to ongoing transmission of the parasite. Because the age disparities in net use seem to be mitigated by sufficient household ownership of nets, school-based net distribution campaign efforts may provide an opportunity to supplement ongoing mass distribution efforts and achieve sufficient net ownership, especially in larger households. Specific information about transmission reservoirs could be incorporated to further promote use, particularly as malaria risk perception has been found to be related to LLIN use in this study and another from Liberia.^18^ Messaging about the importance of using an LLIN to prevent transmission to the mosquito population in addition to providing personal protection has not been developed or tested and is an important area of future research.

An interesting finding from our study was that household members who were not part of the nuclear family of the household head (self, spouse, or child) were significantly less likely to use a bed net in either study site, and that this discrepancy persisted regardless of whether or not the household owned a sufficient number of nets. In fact, the presence of nonnuclear family members who might not be reasonably expected to share a net with the head of household was a common feature of households with an average of one or fewer users per net. The cross-sectional nature of this study limited our ability to determine how transient these household memberships were. People who were not members of the nuclear family comprised a notable 22% of de facto household residents in the highlands and 15.8% in the lowlands. If the household of residence is fluid, it presents a challenge for LLIN campaigns that base net distribution numbers on a static assessment of the number of household members. Efficiently providing LLINs for extended family and other household residents without increasing the number of unnecessary/unused nets may present a significant hurdle to closing the ownership and use gaps in areas that have otherwise achieved high coverage. Furthermore, because having sufficient access did not eliminate the use disparity in this group, qualitative research to understand the nature of complex housing arrangements and reasons that nonnuclear family members were not using bed nets may be needed to help guide new messaging for control efforts to bridge this net use gap.

A key limitation of this study is that it represents net ownership from a single window of time in mid-2015. Maintaining the accomplishments of control efforts requires periodic redistributions as insecticide efficacy degrades over time, LLINs accumulate damage, and population distributions shift. Net care and repair practices influence the level of LLIN effectiveness between distribution campaigns; these behaviors have been evaluated in our study population and discussed in a separate publication.^30^ That analysis found high overall adherence to recommendations, but identified some behaviors, particularly in the lowlands, that may have shortened the effective life of LLINs and could serve as a target for education to maximize LLIN effects between distributions.

Interruption of malaria transmission in highly endemic areas will require both novel tools and innovative approaches with existing tools. This cross-sectional survey found that distribution campaigns had made great strides in LLIN ownership and use in the highlands and lowlands of western Kenya between 2012 and 2015, although gaps remained, particularly in the highlands. Additional resources and efforts are needed to ensure distribution of an adequate number of nets per household, as sufficient access to nets seemed to be the primary limitation to their use in 2015. Distributors should take particular care to supplement net numbers to promote universal access in larger households. Although progress has been made in personal use of LLINs, health messaging may need to incorporate the importance of universal net use to minimize transmission and community risks, with an emphasis on use among school-age children and household members who are not part of the nuclear family.

## Supplemental table

Supplemental materials
