# Supplementary material for: Insufficient Ratio of Long-Lasting Insecticidal Nets to Household Members Limited Universal Usage in Western Kenya: A 2015 Cross-Sectional Study
Source: Am J Trop Med Hyg. 2020 Apr 20;102(6):1328–42. doi: 10.4269/ajtmh.19-0119 (PMC7941599; doi:10.4269/ajtmh.19-0119)
Supplement: Supplementary file 1 [file tpmd190119.SD1.pdf]

Supplemental Table 1. Predictors of households in western Kenya that did not own any nets during a 2015 cross-sectional survey

	Highlands			Lowlands		
	**Total n**	**No nets n (%)**	**Crude OR [1] (95% CI)**	**Total n**	**No nets n (%)**	**Crude OR [1] (95% CI)**
**Households**	643	203 (31.6%)		574	11 (1.9%)	
Household member composition						
**Number of household members**	Mean: 4.26<br>Range: 1-12	Mean: 4.09<br>Range: 1-9	0.95 (0.88 – 1.03)	Mean: 2.92<br>Range: 1-9	Mean: 2.09<br>Range: 1-5	0.70 (0.44 – 1.10)
1 – 2	161	56 (34.8%)	1.00 (ref)	276	8 (2.9%)	1.00 (ref)
3 – 4	199	63 (31.7%)	0.87 (0.56 – 1.35)	190	2 (1.1%)	0.36 (0.08 – 1.70)
5 – 6	177	55 (31.1%)	0.85 (0.54 – 1.33)	85	1 (1.2%)	0.40 (0.05 – 3.24)
>6	106	29 (27.4%)	0.71 (0.41 – 1.21)	23	0 (0.0%)	0 (N/A)
**Child under 5 years living in house**						
No	367	111 (30.2%)	1.00 (ref)	395	10 (2.5%)	1.00 (ref)
Yes	276	92 (33.3%)	1.15 (0.83 – 1.61)	179	1 (0.6%)	0.22 (0.03 – 1.70)
**School-age child (5 – 15 years) living in house**						
No	233	80 (34.3%)	1.00 (ref)	311	7 (2.3%)	1.00 (ref)
Yes	410	123 (30.0%)	0.82 (0.58 – 1.16)	263	4 (1.5%)	0.67 (0.19 – 2.32)
**Perceived malaria severity**						
Low	179	62 (34.6%)	1.00 (ref)	75	3 (4.0%)	1.00 (ref)
Moderate	388	113 (29.1%)	0.78 (0.53 – 1.13)	211	5 (2.4%)	0.58 (0.14 – 2.50)
High	64	23 (35.9%)	1.06 (0.58 – 1.92)	286	3 (1.0%)	0.25 (0.05 – 1.29)
Socioeconomic status						
**Asset ownership quartile**						
Low	165	67 (40.6%)	**2.01 (1.22 – 3.32)**	123	8 (6.5%)	**6.57 (1.37 – 31.50)**
Second	217	66 (30.4%)	1.29 (0.79 – 2.10)	100	0 (0.0%)	0 (N/A)
Third	128	34 (26.6%)	1.06 (0.61 – 1.86)	160	1 (0.6%)	0.59 (0.05 – 6.62)
High	130	33 (25.4%)	1.00 (ref)	191	2 (1.0%)	1.00 (ref)
**Education of the female head of household**						
None or some primary	316	90 (28.5%)	1.00 (ref)	222	5 (2.3%)	1.00 (ref)
Completed primary but not secondary	218	70 (32.1%)	1.19 (0.82 – 1.73)	237	2 (0.8%)	0.35 (0.07 – 1.85)
Completed secondary	50	10 (20.0%)	0.63 (0.30 – 1.31)	47	1 (2.1%)	0.94 (0.11 – 8.27)
No female head or other education	51	29 (56.9%)	**3.31 (1.81 – 6.07)**	51	3 (5.9%)	2.71 (0.63 – 11.74)

	Highlands			Lowlands		
	**Total n**	**No nets n (%)**	**Crude OR [1] (95% CI)**	**Total n**	**No nets n (%)**	**Crude OR [1] (95% CI)**
**Can afford to buy a net if not given one**						
No	198	83 (41.9%)	**1.96 (1.38 – 2.79)**	73	6 (8.2%)	**8.76 (2.60 – 29.49)**
Yes	442	119 (26.9%)	1.00 (ref)	494	5 (1.0%)	1.00 (ref)
Structure characteristics						
**Main building quality [2]**						
Low	52	26 (50.0%)	**2.89 (1.52 – 5.48)**	200	4 (2.0%)	0.66 (0.12 – 3.71)
Moderate	412	132 (32.0%)	1.36 (0.92 – 2.03)	294	5 (1.7%)	0.56 (0.11 – 2.96)
High	175	45 (25.7%)	1.00 (ref)	67	2 (3.0%)	1.00 (ref)
**Vegetation around house**						
None/bare ground	36	15 (41.7%)	1.00 (ref)	191	1 (0.5%)	1.00 (ref)
Short maintained grass	366	112 (30.6%)	0.62 (0.31 – 1.24)	141	4 (2.8%)	5.55 (0.61 – 50.18)
Medium grass and bushes	220	65 (29.5%)	0.59 (0.29 – 1.21)	168	3 (1.8%)	3.45 (0.36 – 33.53)
Long grass and bushes	17	10 (58.8%)	2.00 (0.62 – 6.45)	58	2 (3.4%)	6.79 (0.60 – 76.22)

CI = Confidence interval; OR = Odds ratio

1 Odds that the household does not own any nets.

2 Building quality was based on an index variable calculated from the total number of finished materials used among the walls, floor, and roof of the structure (0, 1, 2, or 3) with an adjustment based on the field worker's rating of household quality on a scale of 1 – 5. Households receiving the median value of the index score (2.5) were defined as 'moderate' quality, while those below and above were respectively defined as "low" or "high" quality.
